# Caregivers’ socio-cultural influences on health-seeking behavior for their wasted children among forcibly displaced Myanmar Nationals and their nearest host communities

**DOI:** 10.3389/fnut.2023.1252657

**Published:** 2023-11-30

**Authors:** Nurun Nahar Naila, Md. Munirul Islam, Aklima Alam, Gobinda Karmakar, Mustafa Mahfuz, Ishita Mostafa, Farhana Sharmin, Mohammad Zahidul Manir, Mayang Sari, Tahmeed Ahmed, Mahfuzur Rahman

**Affiliations:** ^1^Nutrition Research Division, International Centre for Diarrheal Disease Research, Dhaka, Bangladesh; ^2^United Nations Children’s Funds, Dhaka, Bangladesh; ^3^School of Health and Rehabilitation Sciences, The University of Queensland, St Lucia, QLD, Australia

**Keywords:** forcibly displaced Myanmar Nationals, host community, severe wasting, healthcare-seeking behavior, Bangladesh

## Abstract

**Introduction:**

A total of 19% of forcibly displaced Myanmar Nationals (FDMNs) Bangladesh fall within the age range of under five years old, while an average of 1% exhibit severe malnutrition. Cox’s Bazar is the closest host community for FDMNs, with similar traditional culture and religion and shared linguistic, ethnic, and cultural ties.

**Methods:**

A qualitative study was conducted to investigate the impact of socio-cultural factors on the healthcare-seeking behavior of caregivers of critically malnourished children in FDMN camps and neighboring host communities.

**Results:**

The utilization of informal healthcare by caregivers in both populations can be attributed to cultural attitudes, taboos, and peer pressure. The healthcare by practices in the FDMN camps and host towns were primarily affected by household responsibilities, familial assistance in accessing medical services, decisions made by husbands or mothers-in-law, and the availability and accessibility of healthcare facilities. Certain features were identified that prompt caregivers to seek formal treatment in both groups. The efficacy of the treatment was a primary consideration. In instances where conventional remedies and informal treatments proved ineffective in restoring the health of children, others who were invested in their well-being, such as family members and neighbors, advised caretakers to pursue professional medical care.

**Discussion:**

Enhanced caregiver awareness of severe wasting, enhanced healthcare accessibility, and increased community volunteer engagement have the potential to facilitate early identification of severely wasted children and mitigate delays in treatment.

## Introduction

The plight of the Forcibly Displaced Myanmar Nationals (FDMN) community in Bangladesh has been one of the most violent and protracted humanitarian crises in recent history, marked by restricted mobility, denial of citizenship, forcible displacements, and decades-long persecution (1). Violence erupted in Myanmar in the late 1970s, resulting in the displacement of millions of FDMN from its Rakhine state to Bangladesh (2). Currently, more than 1.1 million FDMN are residing in Bangladesh (1) and housed mostly in overcrowded settlements in the Ukhiya and Teknaf Upazilas of the Cox’s Bazaar district. More than 180,000 children under the age of five in this population (3, 4). Around 19% of the FDMN population in Bangladesh (3) are children under the age of five, and around 0.7% of those children are severely malnourished (5). Severe wasting which is a form of malnutrition linked to a mortality rate of 73–187 per 1,000 children per year (6, 7). This condition is life-threatening and requires immediate medical attention (7). However, unfamiliarity with the new setting is very likely to be challenging in healthcare seeking for this vulnerable segment of the population (8).

Health-seeking behavior exhibits variability among many societies and people, influenced by cultural, social, economic, and environmental determinants (9). When examining the health-seeking behavior of FDMNs and the nearest host communities in Bangladesh, it becomes evident that there exist both similarities and differences. They residing in Bangladesh represent a group that has been forcibly relocated, hence giving rise to distinct health-related obstacles associated with their displacement, deprivation of possessions, and psychological distress. In contrast, host communities do not encounter these particular issues (10, 11).

Caregivers of severely wasted children play a crucial role in obtaining appropriate healthcare for their children (12, 13). A significant proportion of female participants from both communities faced the requirement of obtaining permission and financial resources from their male family members to access healthcare services, thereby leading to reduced healthcare accessibility (14). This is even more challenging due to language barriers, cultural differences, and limited access to healthcare services in FDMNs (11). However, language barriers are not as prevalent in the host community, so they do not impede effective communication with healthcare providers like FDMN (15). One of the primary obstacles to accessing healthcare services was the considerable distance between them and the nearest health establishments. The limitation of mobility has been recognized as a hindrance for individuals with disabilities, elderly individuals, and pregnant women due to their advanced age and physical state. Hilly terrain made it difficult for the FDMN population to reach health facilities, which required a 15-min to an hour walk (14).

In addition, FDMN women felt discouraged from obtaining medical assistance from male healthcare providers due to cultural norms that emphasize gender segregation and the need to maintain a modest appearance. Low use of healthcare services among both communities also resulted from several other factors including a lack of trust in the healthcare system and a preference for more traditional forms of treatment (11, 16). This is to be noted that mistrust of FDMNs and host communities about modern medicine is a big challenge for healthcare practitioners. Due to their beliefs in spiritual and supernatural causes of illness, they favor traditional medicine over modern medicine (17). Although the health perspectives of the two communities may be similar, their traditional healing practices are distinct; these cultural distinctions may not have been acknowledged and respected in health care services (11). Furthermore, the COVID-19 pandemic has increased the FDMNs’ fear and mistrust of medical professionals, leading them to assume that if they are admitted to the hospital with COVID-19 infection, they will be killed to prevent the virus from spreading (17).

Though key humanitarian assistance programs supported by United Nations (UN) agencies and implemented by non-government organizations (NGO), such as household food rations, ready-to-use therapeutic food (RUTF), fortified blended foods, and micronutrient powders, have expanded greatly (18); the FDMN population remains largely unacquainted with health services (19). In contrast to that, since 2017, approximately 46 NGOs and UN agencies, and the Bangladesh government have been helping host communities with education, food security, gender-based violence, health, shelter, nutrition, protection, and WASH (6). The FDMNs’ protracted stay has limited resources for the host population near the camps. Stress on resources causes other host community difficulties (7). The rapid influx of FDMNs into Bangladesh and rising commodity prices have hurt host communities (8). This influx reduced host community income and work prospects and negatively impacted diet diversity, putting children at risk of malnutrition (7).

The convergence of health-seeking behavior among FDMNs and the host community in Bangladesh presents a multitude of advantages. However, it is imperative to acknowledge and tackle potential obstacles, including cultural disparities, linguistic limitations, and the necessity for culturally sensitive healthcare provisions that cater to the distinct requirements of both groups. Therefore, we explored caregivers’ sociocultural behavior influence on seeking treatment for their wasted children among Forcibly Displaced Myanmar Nationals and their nearest host communities.

The study findings will provide valuable assistance to the health sector in determining how to effectively deliver appropriate, culturally sensitive, and sufficient resources for severely malnourished children experiencing wasting.

## Methods and materials

### Study design

We used a qualitative approach to perform the study. The study was exploratory and gave us a better understanding of the healthcare-seeking behavior of the caregivers of the severe wasted children among the FDMN communities and their nearest host communities in Cox’s Bazar, Bangladesh. Children of the host community have been treated at different types of healthcare facilities, including government hospitals, private doctors, and nutrition centers; whereas FDMN children get treatment from Integrated Nutrition Facilities or Health Posts situated in every camp (14, 18). Some of these facilities provided free services (only for FDMN), while others charged fees. Few children from FDMN camps were admitted to stabilization centers, and some were referred to hospitals for further examination and testing, such as chest X-rays and skull X-rays.

### Study participants

We included caregivers of the children of 6–59 months of the FDMN and their nearest host communities in the Teknaf sub-district of Cox’s Bazar district, Bangladesh. Caregivers were described as mothers or other family members who took care of the children most of the time.

We purposively selected the caregivers whose children had recently (within 6 months prior to data collection) received or were getting, inpatient or outpatient treatment for severely wasted children in any healthcare facilities in the study areas. These caregivers were found using records from the integrated nutrition facilities (INFs) in the camps for FDMNs and from any healthcare facilities in Teknaf, Cox’s Bazar for host communities. We conducted a total of 17 IDIs (In-Depth Interviews) (8 from the host communities and 9 from the FDMN refugee communities) considering the principles of data saturation.

### Study site

The research was conducted in FDMN Camps 25 and 27 and their nearest host communities in the Teknaf sub-district of the Cox’s Bazar district. The selection of research areas was determined by the emergency in the aforementioned region. Due to a nutritional emergency, the site was selected where, later on, the intervention of the effectiveness trial of locally produced therapeutic food would be rolled out among the children of the FDMNs suffering from severe wasting without complications.

Camp 25 and Camp 27 collectively contain four health centers. The management and operation of these health centers are divided among many organizations. Specifically, the International Rescue Committee and Terre des Hommes are responsible for the management and operation of health centers in these camps, under the oversight of Save the Children. Additionally, the United Nations Children’s Fund (UNICEF) manages the Integrated Nutrition Facility within these camps. In aggregate, these health centers encompass a collective sum of 50 healthcare practitioners. The total population of both camps amounts to 25,964 individuals. As a consequence, there exists a ratio of one health worker for 519 individuals in the population. The mean distance between the residences of participants and the closest health institution is 1.5 kilometers. Utilizing healthcare services incurs no associated expenses, including expenditures related to travel and personal donations. The inhabitants of the camp access the services on foot. There is no expenditure associated with utilizing healthcare services, encompassing travel costs and personal contributions. The residents of the camp receive their services by walking.

The other site of the study was in Hnila Union, a 65,000-person host community. Hnila Union has six health centers. Three community clinics and one Union Health Complex are in the Union. Teknaf Upazila Health Complex and Cox’s Bazar District Hospital are the other two hospitals outside the Union. Residents rarely visit the district hospital unless they have a serious illness. In the three community clinics, Union Health Complex, and Teknaf Upazila Health Complex, 119 healthcare providers work. Health workers are 1:2100 to the population. Participants average 4.5 km from their homes to neighborhood clinics and the Union Health & Family Welfare Center (UH&FWC). The average distance to Upazila Health Complex is 20 miles, and Cox’s Bazar District Hospital is 66 kilometers. Healthcare costs, including travel and out-of-pocket charges, vary per facility. On average, participants pay 50 Taka to reach a Community Clinic, 120 Taka to the Upazila Health Complex, and 450 Taka to the District Hospital.

Most of the population is Bengali and works as laborers from lower-middle-class backgrounds. Most people speak Bengali and are Muslim.12.8% of the population suffers from global acute malnutrition (Weight-for-height z score < −2.0) and 7.3% from moderate acute malnutrition (Z score < −2.0 & > −3.0). In 5.5% of the population, severe acute malnutrition (Weight-for-height z score < −3.0) is seen.

### Data collection

Data collection is a crucial step in qualitative research that involves gathering information from various sources to answer research queries. We gathered both verbal and written informed consent from all the participants before the interviews. Data collection occurred from 15th March to 8th August 2022. Four experienced Field Research Assistants with training in Social Science were engaged in data collection under the supervision of an investigator (MR). Two were female, while the other two were male. They were recruited from the surrounding areas so that they could speak and comprehend the participants’ dialect (or local language). Additionally, two investigators (MR and NNN) visited the study sites and received information from the interviewers, and provided necessary feedback for further exploration or clarity of data. Before conducting interviews, the data collectors visited the participants’ homes and established rapport with them.

We conducted in-depth interviews (IDIs) with the purposively selected caregivers of the children who had recently received or were currently receiving treatment for SAM in INF and community clinics to gain in-depth insights and understand caregivers’ experiences of health-seeking behavior for their wasted children. The roster of children was obtained from the facilities and/or by visiting the homes of the children’s primary caregivers. The participants were purposefully selected to maximize variation in terms of education, age, number of offspring, etc. All IDIs were conducted at the level of the household. The interview was conducted using a topic guide (Supplementary Table S2) as a guide. On average, each IDIs lasted 86 min. We conducted a total of eight IDIs from the host communities and nine IDIs from the FDMN communities following the data saturation principle.

### Data analysis

In this study, we adopted the definition of a traditional healer from the World Health Organization as a person who does not have formal medical training but is recognized (by the local community) as being qualified to provide health care using animal, plant, and mineral substances and specific alternative methods based on the social, cultural, and religious background that includes the knowledge, attitudes, and beliefs that have been widely accepted in the community regarding physical, mental, and social well-being as well as the underlying causes of sickness and impairment (20). The typology of formal and informal healthcare providers have been conceptualized based on their training (received formally recognized training with a defined curriculum from a government or academic institution or not), and registration (registered with any government regulatory body or not) (19).

After the interviews were completed, the recordings were transcribed and summarized within 2–3 days (depending on the duration of interviews or discussions) by experienced qualitative researchers. To become familiar with the data, the transcripts and summaries were reviewed very carefully. Peer briefings were held, and field notes were given to the investigators by the data collectors, to gain feedback on what concerns the investigators needed to look into further. The investigator gave timely input on such issues so that the data collectors might study those issues in greater depth during the subsequent interviews or discussions. In this iterative approach, initial analysis was performed while data were still being collected.

In addition, we used a qualitative technique to establish the tenet of credibility in trustworthiness to verify the findings of our research. During the process of qualitative technique, our research team went back to the selected study participants with their transcripts and read aloud the transcripts to the respective participants. They then asked the participants for their comments on whether or not the transcripts were justifiable from their points of view. During the process of member checking, the members of the research team also tried to gain a better understanding of any difficulties (if any) that required additional clarifications.

In the end, we examined the content. Two Research Assistants extracted the condensed meaning units from the raw data, tabulated them, and then finalized the meaning units by checking their work against each other and with each other. After that, data collectors carried out the coding, then the patterns were identified and the findings were interpreted. The results of the study have been compiled and presented in a checklist format that adheres to the consolidated standards for reporting qualitative research (COREQ) (21) (Supplementary Table S3).

## Results

The results in this paper have been presented based on thematic areas generated from the study. The themes included different health-seeking behaviors such as home remedies and foods, traditional and informal healthcare seeking, formal healthcare seeking, and the factors triggering the caregivers to seek care from one contact to another contact for healthcare. Until severe wasting was diagnosed or identified in formal healthcare facilities, the caregivers of the children sought care from different contacts (from traditional to informal to formal, or simultaneously from multiple contacts) perceiving the severity of problems or diseases.

Although the participants were from two different communities-FDMN and their host community, their characteristics to some extent varied in terms of their age, education, and number of family members. Some basic characteristics of the interviewees have been given in Table 1.

**Table 1:** Basic characteristics of the study participants.

Characteristics	Caregivers from FDMN	Caregivers from host communities
Number of participants	9	8
Child’s age (average in months)	22.6 (Range 7–52)	23.8 (Range 11–42)
Mother’s age (average in years)	26.4 (Range 20–33)	25.8 (Range 19–32)
Mother’s years of schooling (average)	3 (Range 0–8)	4 (Range 0–10)
Mother’s occupation	Housewife (9)	Housewife (6), Tailor (1)
Father’s age (average years)	28.8 (Range 22–35)	29.0 (Range 20–35)
Father’s years of schooling (average)	3 (Range 0–10)	5 (Range 0–14)
Father’s employment status (Employed in number)	Employed (5), Un-employed (4)	Employed (7), Un-employed (1)
Average number of household members	5 (Range 3–8)	6 (Range 3–12)
Average number of children’s below 5 years of age	2 (Range 1–3)	1 (Range 1–2)
Average family income per month (in BDT)	6000.0 (5000–7,000)	14266.6 (6000–30,000)

### Home remedies and foods

The majority of caregivers for severely malnourished children from both the FDMN and host communities initially relied on home remedies before their children were formally diagnosed as severely malnourished by healthcare professionals. Home remedies include various food options, including homemade meals consisting of rice, pulses, potatoes, and vegetables, commercially available infant formula, rice powder mixed with sugar or misri, soft rice, suji, khichuri, fruits, eggs, fish, and vegetables. However, these caregivers initially thought their children’s weight loss was due to a lack of food, so they fed them alternative foods. We noticed a mother within the FDMN community who resorted to selling her earrings to purchase infant formula for her baby.

“My boy's father brought 3 annas of gold earrings for me, he says that even it takes to sell the gold to feed the child, he will do that. That's why we sold it. We used to buy lactogen for six hundred and fifty taka. I have fed the child by selling gold.”

A mother who perceived that her child was getting thin and sick not for wasting, mentioned:

“When the child became thin and sick, I fed him blended rice with lactogen. Also, I didn't know that the child needed treatment for undernutrition, though I tried a lot for the treatment of sickness.”

Another mother added:

“… I was giving my child rice flour. I used to mix the rice in the dheki (a traditional manually operated equipment used to prepare powder) before frying and storing it in a bottle. Then I prepared the child's food with misri.”

We found two mothers from both communities who tried home remedies for their children before their children were diagnosed as severely wasted. A child in the host community often suffered from common illnesses such as cough and cold, his mother made the child drink warm mustard oil with garlic lemon, and tea.

*“I used to prepare a solution of mustard oil, lemon juice, and onion powder before feeding it to my child. I forced my child to consume tea. I massaged his entire body with mustard oil before giving him a shower. Then I pour oil on him again and dry him in the sun*.”

A mother from the FDMN community tried “*Tula Oushod”* (made of neem leaves and various other leaves and roots) for her *Leda* (severely wasted) child.

### Traditional healings

Six mothers of FDMN camp children sought treatment from Vaidya and Moulana healers. Religious leaders are revered in FDMN culture and influence behavior, including child healthcare (22). Their beliefs and respect are shared by the host community. Children in both situations sought treatment early on. They can visit the Moulana anytime without an appointment. Due to their disease treatment inexperience, individuals need recurrent visits. Per visit, 300 to 2000 taka ($18 to $20) are needed to finish the process.

We found two more children with an evil spirit (jinn-chumma dio paise) who were taken to Moulana and given enchanted oil, amulets, and advice to bury a burned egg. Traditional healers Moulana and Vaidya advised three children to drink charmed water and rub mustard oil. One caregiver was advised by a Vaidya to draw an eyeball on paper to escape the bad spirit.

“I was told in the community that evil spirits possessed my child and they were sucking blood of him and that’s why I have gone to the Vaidya 3 times. I drew an eyeball on a paper and then I took a handful of rice on the paper. Then I made a boat with the paper and swept the body of my child with that boat and finally I threw that away.”

Some parents read religious holy book verses (Surah) and blew air at the children. These remedies were believed to cure bad spirits-caused sobbing, lack of appetite, and convulsions. Even though symptoms returned, these therapies were questionable. Traditional healing is appropriate and shorter. Mothers find solace in not having to leave domestic duties unfinished.

Three caretakers of wasting children visited Vaidya and one visited Maulana after being informed by neighbors or elderly family members in the host communities. A caregiver’s cultural beliefs may lead her to see both a traditional healer and a doctor. A carer who attended a private practitioner also visited Vaidya because the evil spirit (Jinn) was sucking her child’s blood and thinning him. One of the three caretakers visited Vaidya numerous times but stopped after her child did not recover. She said:

“I took him to Vaidya for treatment. He gave nothing but amulets, enchanted water, and mustard oil. He advised me to tie the amulets, wash the body with enchanted water, and then massage the body with enchanted oil. I went there three times in 1-month intervals. Every time he took 200 taka for this. I have to sell some of my food rations to pay the fee. But my child didn’t recover after all of this.”

### Informal and alternative healthcare

In both communities, children were given various informal treatments from pharmacies, including saline and syrup for fever, cough, common cold, and diarrhea. Medicines including vitamin syrups were frequently bought from pharmacies. They need to purchase the required medicines from the pharmacy. In severe instances, the child received an injection and syrup at the pharmacy for diarrhea and was asked to return for another injection the following day. Homeopathy was also used for invasive diarrhea, cough, and cold. Seeking treatment from pharmacies and homeopathist were triggered by family members themselves. Homeopathy is pseudoscientific; the practitioners of homeopathy are called homeopaths and they believe that the drug that causes sickness symptoms in healthy persons can alleviate comparable symptoms in sick people. Sometimes homeopathies and pharmacies were the first contact point for treatments due to the long queues in the primary formal healthcare points in the FDMN community. A caregiver, whose child was suffering from severe wasting and having diarrhea, said:

“Once my child had severe diarrhoea. At first, his father took him to a hospital. There was a long queue and the child frequently purged; he had to change clothes repeatedly and the child became weak. The child’s father got annoyed and left the place and brought the child to a pharmacy for treatment.”

The main reason host communities sought care from informal or alternative healthcare providers was their accessibility and availability. Most limitations from spouses and mothers-in-law prevent mothers from seeking formal care outside the home. Many FDMN households have more than 5–6 children. Due to the pressure of caring for other children, mothers typically avoid formal care. They can easily obtain and use informal remedies like pharmacy and homeopathic drugs. Lack of transportation or great distances to official healthcare institutions also hindered treatment. Such caregivers preferred informal or alternative therapies. Peer pressure and neighbors also drove informal healthcare use.

### Formal healthcare

INF and community clinics were the first formal contacts for wasting children in FDMN and host communities, respectively. Since community nutrition volunteers screened wasted children in FDMN camps, caregivers were more likely to seek formal healthcare facilities (INFs). A caregiver from the FDMN camp mentioned:

“The Community Nutrition Volunteer visited my house and took my child’s measurements. They said that my child was undernourished and he needed treatment. After that, I took him there (INF) and received lalpushti (RUTF). After taking lalpushti he was doing better than.”

Of the nine FDMN camp caretakers interviewed, two initially attended formal healthcare facilities (INFs). One visited INF again after pharmacy services.

None of the eight host community caregivers interviewed visited the clinic first. However, two carers first saw private practitioners.

In wasting treatment, most caretakers in both groups received certified treatment for diarrhea, pneumonia, cold cough, sores, blisters, falls, and fever. Some families visited many hospitals for their children’s health. Both groups’ parents cannot receive healthcare for their children due to domestic duties and family head consent. A mother said from the host community and FDMN accordingly:

“I had always taken permission from my mother-in-law’ and “We fought for not telling my husband that I am going to take Pushti.”

Financial and transportation constraints plagued families seeking child care in both locations. However, FDMN caregivers sometimes encounter language problems when seeking care from non-INF physicians. Some families borrowed money to pay for medical expenditures and traveled far to get care. Families had trouble understanding diagnosis and treatment options because healthcare staff spoke a foreign language.

In both communities, we revealed some triggering factors that eventually led the caregivers to switch to formal treatment options. One of these factors was treatment outcome. When the health condition of children did not improve despite seeking assistance from traditional healers or opting for informal treatments to ensure child wellbeing, concerned family members and neighbors advised the caregivers to seek formal healthcare care.

### Challenges of or barriers to utilization of services for wasted children

The provision of care for severe malnourished children faced numerous obstacles and limitations in both FDMN camps and host communities. Within the confines of the camps, there existed a situation where husbands displayed hesitancy in granting permission for their wives to be admitted to Stabilization Centers (SCs) despite the presence of acute malnourishment and associated difficulties in their children. The hesitance stems from the challenges associated with providing care for additional children inside the household in the absence of the primary caregiver. Furthermore, it should be noted that SCs enforced a policy that disallowed males from staying overnight, thus necessitating the presence of female caregivers to remain with the children. This additional need further exacerbated the complexities of the situation. Nevertheless, as a result of domestic responsibilities and insufficient familial assistance, these female caregivers frequently exhibited reluctance to remain at the SCs. In the context of host communities, caregivers originally sought assistance for extremely malnourished children at community clinics (CCs). In cases where a child has been identified with severe malnutrition and associated difficulties, it is customary for them to be referred to Upazila Health Complexes (UHCs). This context presented comparable difficulties, encompassing domestic obligations, insufficient assistance for other offspring, and resistance from spouses concerning overnight visits.

The summary has been displayed in Supplementary Table S1.

## Discussion

This qualitative research explored how caregivers of malnourished children among the FDMN and their neighboring host communities in Bangladesh sought healthcare from various providers. The findings of the study revealed a multitude of elements that exerted an effect on the decision-making process of individuals when they were seeking healthcare services.

The FDMN’s nearest host community in Cox’s Bazar is more conservative in culture and religion than the rest of Bangladesh and shares linguistic, ethnic, and cultural similarities. Nearly 40% of Upazillas near Teknaf and Ukhiya camps are poor, compared to 24% overall. After considerable migration, their health-seeking habits changed (16). In host societies, family members equate filth and impurity with childbirth and segregate the mother and baby to protect them from disease and evil spirits (23). Cultural, religious, and patriarchal views limited FDMN women’s reproductive health choices. As part of their traditional medical practices, many FDMNs use herbal medications and spiritual healing (19). In both host communities and FDMN considered informal healthcare providers including neighborhood drug store, Vaidyas, and moulabi is the most accessible. Consistent with the results of another study, it was shown that they visit informal healthcare providers when they become sick (14). In both of these communities, we discovered both beliefs and misconceptions concerning the severe malnutrition of the children. It is important to spread the positive beliefs while working to eradicate harmful misconceptions, as the later may be one of the contributing causes that lead to child wasting (24). Because of this, some people wait longer than necessary to seek medical treatment, which can make their existing health concerns much worse. Various factors, such as cultural differences, financial constraints, and the perception that a disease or its symptoms would naturally resolve, contribute to the decision of certain individuals to forgo official medical intervention (25).

Every day, Bangladeshi FDMNs face malnutrition and a lack of healthcare (26); this phenomenon is not uncommon in other cultures as well (27). Low income (28), illiteracy, and cultural beliefs that prohibit medical treatment exacerbate these issues. This study found that many FDMN and host communities practice spiritual healing and herbal medicine for their children. The Haider et al. study validates our findings, showing that herbal and spiritual treatments, imams, and religious leaders are often sought instead of medical care (11). This dependence on traditional techniques and spiritual healing suggests focused measures to raise knowledge of the benefits of professional medical care and reduce delays in obtaining treatment for severely wasted youngsters.

In both communities, caregivers sought informal treatment for diarrhea, fever, cold, and cough from pharmacies, according to our study that aligns with the research findings that were conducted in African settings, Nigeria (29). This study identified several barriers to seeking formal treatment, including limited accessibility to and availability of formal healthcare facilities, transportation challenges, and long distances. Furthermore, family members and neighbors played a role in influencing the decision to seek care from informal or alternative healthcare providers. Another study also has reinforced our findings in the context of FDMN and host populations in that they seek services from pharmacies, particularly for illnesses like fever, diarrhea, and common cold or cough (28, 30).

Parents, usually women from the host community, know the value of facility-based care but cannot actively seek it due to societal traditions since their community rejects hospital-based delivery (31). Additionally, FDMN camp residents must deal with healthcare facilities. FDMN refugees report a lack of medical facilities and qualified medical staff in several camps (32). Because of this, many FDMNs travel far to get medical care, which can be difficult. Globally, this phenomenon is not infrequent, and prior studies have also demonstrated that transportation expenses play a significant role in accessing formal healthcare services (33).

The findings of this study provide valuable insights into the challenges faced by caregivers in seeking formal healthcare for wasted children among the FDMN communities and their host communities. The initial formal contact for wasted children differed between the two communities, with the integrated nutrition facilities (INF) being the primary point of contact for the FDMN camps and the community clinic serving as the initial point of contact for the host communities. One significant observation was that caregivers in both communities were often unaware that their children required treatment for wasting. This lack of awareness necessitates improved health education and awareness campaigns targeting caregivers to enhance their understanding of wasting and its associated risks. The study highlights a widespread deficiency in health literacy, specifically in relation to the management of common ailments like as diarrhea. This finding is of significant significance within the context of the study’s environment (33). Nevertheless, the enhancement of consciousness regarding the significance of health literacy (34) and the acquisition of dietary knowledge among carers may contribute to the attainment of the aforementioned advantage (35). Language barriers were identified by FDMN caregivers seeking medical care outside of INFs. Communication difficulties made healthcare providers’ diagnoses and treatment options unclear to caregivers. Research suggests using interpreters and translators in healthcare to help different populations understand medical information (34, 35).

Our investigation identified triggers that lead caregivers to formal therapy. One was the treatment result. When traditional healers or informal remedies failed to improve wasted children’s health, concerned family and neighbors recommended caregivers to seek formal healthcare. Social support and community impact decision-making about formal care for wasting children.

As a result of their forced displacement and the cultural beliefs to which they adhere, the FDMNs in Bangladesh have a wide range of difficulties in seeking care for their wasted children. On the other hand, the host communities who are also suffering from resource constraints for hosting the FDMN communities, also need support for seeking timely and appropriate care for their wasted children. Our study suggests that increased engagement of community volunteers to caregivers can be crucial in the early detection of severely wasted children. Therefore, it will reduce the delay in treatment-seeking in both the FDMN and their nearest communities.

### Limitations of the study

There are various limitations inherent in our investigation. The study design employed in this research facilitated the identification of primary themes across participant groups. However, it should be noted that our design does not provide a means to quantify the frequency of these themes or establish a hierarchical ranking based on their level of significance. Furthermore, it should be noted that due to resource constraints, our study was unable to recruit a larger number of focus groups representing each demographic stratum, hence limiting our capacity to thoroughly compare responses across subgroups. While our efforts were focused on creating comprehensive interview guidelines that may potentially generate applicable replies in other contexts, it is important to acknowledge that the cultural nuances inherent in this study may not necessarily be transferable to different settings.

### Recommendation of the study

The findings obtained from this study will be utilized to inform a further quantitative investigation, which will be done and modified across several contexts to enhance contextual validity.

## Conclusion

Findings indicate that in both communities, cultural beliefs, household affordability, and accessibility to healthcare facilities prompt caregivers to seek care from various categories of healthcare providers, as is typically observed in a pluralistic healthcare system. Therefore, it is essential to implement solutions based on the presented findings and recommendations, with the potential for further investigation to mitigate future challenges.

## Data availability statement

The raw data supporting the conclusions of this article will be made available by the authors, without undue reservation.

## Ethics statement

The studies involving humans were approved by the Ethical Research Committee under the Institutional Review Board of icddr,b. The studies were conducted in accordance with the local legislation and institutional requirements. Written informed consent for participation in this study was provided by the participants’ legal guardians/next of kin.

## Author contributions

NN and MR conceptualized the study, drafted the original manuscript, and revised the final version of the manuscript. MR, AA, GK, and IM had contributed to data analyses. MI, MuM, FS, MZM, MS, and TA critically reviewed the manuscripts. All authors contributed to the article and approved the submitted version.
