# Supplementary figures and images for: Caregivers’ socio-cultural influences on health-seeking behavior for their wasted children among forcibly displaced Myanmar Nationals and their nearest host communities
Source: Front Nutr. 2023 Nov 30;10:1252657. doi: 10.3389/fnut.2023.1252657 (PMC10720355; doi:10.3389/fnut.2023.1252657)

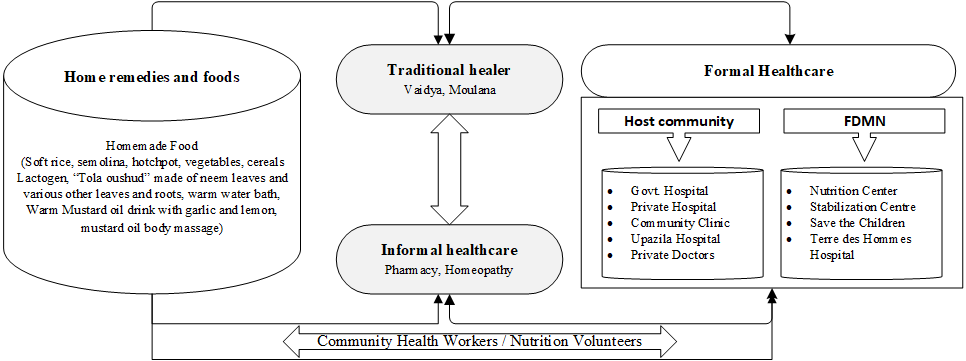

Supplement: Supplementary file 1 [file Image_1.tif]
