# Supplementary material for: Caregivers’ socio-cultural influences on health-seeking behavior for their wasted children among forcibly displaced Myanmar Nationals and their nearest host communities
Source: Front Nutr. 2023 Nov 30;10:1252657. doi: 10.3389/fnut.2023.1252657 (PMC10720355; doi:10.3389/fnut.2023.1252657)
Supplement: Supplementary file 2 [file Table_1.docx]

**Supplementary Table 1:** **Health-seeking behavior and practices for both FDMNs and host communities**

|  | **Quotes** | **Findings** |
| --- | --- | --- |
| **Home remedies and foods** | *“My boy's father brought 3 annas of gold earrings for me, he says that even it takes to sell the gold to feed the child, he will do that. That's why we sold it. We used to buy lactogen for six hundred and fifty taka. I have fed the child by selling gold”.* | The father of the son acquired gold earrings for the mother, which were then sold to obtain formula milk (specifically, Lactogen) for a sum of 650 takas, to nourish the infant. |
|  | *“When the child became thin and sick, I fed him blended rice with lactogen. Also, I didn't know that the child needed treatment for undernutrition, though I tried a lot for the treatment of sickness”.* | The kid showed symptoms of malnourishment, including weight loss and illness. In an attempt to alleviate the child's condition, the mother provided a diet consisting of blended rice supplemented with lactogen. However, it is important to note that the mother was uninformed of the child's concurrent need for treatment specifically targeting undernutrition. |
|  | *“… I was giving my child rice flour. I used to mix the rice in the dheki (a traditional manually operated equipment used to prepare powder) before frying and storing it in a bottle. Then I prepared the child's food with misri”.* | The mother provided sustenance to her child by utilizing a traditional method of manually grinding rice flour using a manually powered equipment known as a "Dheki". Subsequently, the flour was fried and stored in a container. The mother then proceeded to prepare the child's meal by incorporating a sweetening agent called "Misri". |
|  | *“I used to prepare a solution of mustard oil, lemon juice, and onion powder before feeding it to my child. I forced my child to consume tea. I massaged his entire body with mustard oil before giving him a shower. Then I pour oil on him again and dry him in the sun”.* | The caregiver applied a mixture consisting of mustard oil, lemon juice, and onion powder, compelled their offspring to consume tea, conducted a massage using mustard oil before bathing, and subjected the child to sun exposure as components of their caregiving regimen. |
| **Traditional healings** | *“I was told in the community that evil spirits possessed my child and they were sucking blood of him and that’s why I have gone to the Vaidya 3 times. I drew an eyeball on a paper and then I took a handful of rice on the paper. Then I made a boat with the paper and swept the body of my child with that boat and finally I threw that away”.* | The parents sought the services of a traditional healer, known as a Vaidya, on three occasions in response to a prevailing community belief that their child was being afflicted by malevolent spirits. The healing process involved a ritual that entailed drawing an eyeball on a piece of paper, placing rice upon it, constructing a paper boat, and subsequently sweeping the child's body with it. However, the efficacy of this treatment remains uncertain. |
|  | *I took him to Vaidya for treatment. He gave nothing but amulets, enchanted water, and mustard oil. He advised me to tie the amulets, wash the body with enchanted water, and then massage the body with enchanted oil. I went there three times in 1-month intervals. Every time he took 200 taka for this. I have to sell some of my food rations to pay the fee. But my child didn’t recover after all of this”.* | Parents sought treatment from a traditional healer (Vaidya) for their child, who provided amulets, enchanted water, and mustard oil treatments for three visits at 200 takas each, but the child did not improve. |
| **Informal and alternative healthcare** | *“Once my child had severe diarrhoea. At first, his father took him to a hospital. There was a long queue and the child frequently purged; he had to change clothes repeatedly and the child became weak. The child’s father got annoyed and left the place and brought the child to a pharmacy for treatment.”* | The parents of the child sought medical assistance from a traditional healer, commonly known as a Vaidya. The Vaidya prescribed several remedies including amulets, enchanted water, and mustard oil treatments during three separate visits. Each visit incurred a charge of 200 taka. However, despite these interventions, the child's condition did not show any signs of improvement. |
| **Formal healthcare** | *“The Community Nutrition Volunteer visited my house and took my child’s measurements. They said that my child was undernourished and he needed treatment. After that, I took him there (INF) and received lalpushti (RUTF). After taking lalpushti he was doing better than.”* | The residence was visited by community nutrition volunteers who observed that the child was experiencing undernutrition. Following the administration of lalpushti, there was a noticeable enhancement in the child's overall health. |
| **Challenges of or barriers to utilization of services for wasted children** | | |
| **FDMN** | *“When I try to keep my other kids at home, they either get themselves stuck in the drain or make a huge mess of the entire house. Sometimes thieves comes over and steal our preserved foods. The entire service in the Pushtikhana lasted around an hour. There is no one available to watch the kids while I work. We also have to wait for hours and hours in the Pushtikhana before we can use the service.”* | To get the nutrition RUTF, the mother has to bring her children along, and it takes about an hour to reach the nutrition center. This means she has to leave her other children at home with no one to watch over them. Leaving the children at home also makes the house messy, and there's a risk of thieves coming in when the kids are outside. Plus, there's a long line at the nutrition center, so she has to stand for a long time, which is hard for her. |
| **Host community** | *“It appears that transportation is a major issue. Bringing kids on a Tomtom is challenging. It's costly and sacred, which is a bad combination. It costs 40 taka one way and another 40 taka to return. In addition, the entire service requires an additional two hours.”* | They must travel to the nutrition center with their children. To reach there they use a rickshaw called "tomtom," which can be quite difficult to ride with a baby. The journey takes approximately one hour and costs about 80 takas for the round-trip |
| **Suggestions to make the services more available, accessible, and affordable** | | |
| **FDMN** | *“It would be wonderful if CNVs could visit our homes and hand us each a Pushi. It might end up saving us both time and money.”* | Considering this concern, she suggested that it would be easier if the volunteers went door to door and distributed RUTF. This would have reduced the suffering of the mother and the children. |
| **Host Communities** | *“Either bring the goods to us, or construct an office solely for us to use.”* | It would greatly improve the situation if RUTF could be delivered to their homes or if an office could be established closer to their residences. |
