# Supplementary material for: Caregivers’ socio-cultural influences on health-seeking behavior for their wasted children among forcibly displaced Myanmar Nationals and their nearest host communities
Source: Front Nutr. 2023 Nov 30;10:1252657. doi: 10.3389/fnut.2023.1252657 (PMC10720355; doi:10.3389/fnut.2023.1252657)
Supplement: Supplementary file 3 [file Table_2.docx]

**Supplementary Table 2: Guideline for in-depth interviews with the caregivers of the children who have received or are currently receiving services for their wasted children**

| **Theme and Sub-**  **Theme** | **Results** | |
| --- | --- | --- |
| **Domain 1: Basic characteristics of the caregivers of the children who have received or are currently receiving services for their wasted children** | | |
|  | **FDMN** | **Host communities** |
| **Theme:**  Theme: Caregivers of Children with wasted conditions  *The input focuses on the basic characteristics of caregivers who are responsible for children with wasted conditions*  **Sub-Theme:**  Demographic profile of caregivers and children  *The input provides information about the characteristics of the caregiver and the children, including their children* | **Participants (Caregivers):**   - Number of participants: 9 - All are mothers   **Child Information:**   - Child's average age: 22.6 months   **Mother's Information:**   - Mothers average age: 26.4 years - Mothers average years of schooling: 3 years - Mothers occupation: housewife (9)   **Father's Information:**   - Fathers average age: 28.8 years - Fathers average years of schooling: 3 years - Fathers employment status: Employed (5), Unemployed (4)   **Household Information:**   - Average number of household members: 5 - Average number of children below 5 years of age: 2 - Average family income per month (in BDT): 6,000 | **Participants (Caregivers):**   - Number of participants: 8 - All are mothers except one grandmother   **Child Information:**   - Children average age:23.8 months   **Mother's Information:**   - Mothers average age: 25.8 years - Mothers average years of schooling: 4 years - Mothers occupation: housewife (6), tailor (1)   **Father's Information:**   - Fathers average age:29 years - Fathers average years of schooling: 5 years - Fathers employment status:   Employed (7), Unemployed (1)  **Household Information:**   - Average number of household members: 6 - Average Number of Children Below 5 Years of Age: 1 - Average Family Income per Month (in BDT): 14266.6 |
|  |  |  |
| **Domain 2: Health seeking behavior** | | |
| **Theme:**  Seeking care of a child  **Subtheme:**  Recognizing the need for seeking care for a child | When an infant shows signs of weight loss, reduced intake of breast milk, refusal to consume solid meals, excessive crying, decreased activity levels, or displays symptoms such as fever, cough, cold, or diarrhea, parents or caregivers recognize the significance of obtaining medical intervention for the child. | |
| **Theme:** Taking care of a sick child  **Sub Theme:** Triggers and healthcare seeking  *The input discusses the factors that led to seeking care, including the first and second contacts with healthcare* | Home remedies and informal healthcare were used by Rohingya refugees and host communities before formal healthcare. Some caregivers believed their severely wasted children were spiritually ill and sought treatment from traditional healers (Kabiraz or Baidya). In Rohingya refugee communities, outreach workers' visits and MUAC measurements prompted caregivers to seek INF care for their severely emaciated children. In both Rohingya refugee and host groups, caregivers sought care for their severely wasted children when friends and relatives saw and encouraged them, and when their children had diarrhea or fever. Rohingya refugee children's caregivers visited Pushtikhana (INF) or health-posts and even private doctors' offices outside of camps. However, host community caregivers attended neighborhood clinics, Upazila health complex, and icddr,b hospital, Teknaf. | |
| **Domain 3: Challenges of or barriers to utilization of services for wasted children** | | |
| **Theme:** Parental satisfaction with child services  **Sub Theme:** Evaluation of service accessibility and provider behavior  *The input focuses on two main aspects: the behavior and attitudes of the service providers, and the accessibility of the services. The individual is asked to share their feelings* | In Rohingya refugee camps and host communities, severely wasted children had trouble using services. Some men refused to let their wives stay in Stabilization Centers (SCs) for chronically malnourished children with difficulties in the camps. This reluctance resulted from challenges caring for other children at home without the primary caregiver. SCs did not allow males to stay overnight, thus female caregivers had to watch the youngsters, complicating issues. These female caregivers were often unwilling to stay at SCs due to household tasks and family support issues. In host communities, caregivers sought help at CCs for severely wasted youngsters. Children with severe malnutrition and problems were referred to Upazila Health Complexes. Here, home duties, lack of help for other children, and spouse opposition to overnight visits were common. | |
| **Domain 4:** **Suggestions to make the services more available, accessible and affordable** | | |
| **Theme:** Improving Service Availability, Accessibility, and Affordability  **Sub Theme:**  To make the service more available, it is suggested to increase its availability by expanding its reach to a wider audience. This can be achieved by establishing more service centers or branches in different locations, especially in areas with | FDMN camp and host community caregivers have trouble getting assistance for critically impoverished children. Caregiver recommendations included giving Ready-to-Use Therapeutic Food (RUTF) and offering height, weight, and apprtitr checks in camp blocks. This would lessen Pushticentre (INF) crowds and save time.  In host communities, caregivers said distance prevented severely wasted youngsters with difficulties from receiving healthcare. Teknaf Upazila Health Complex (UHC) was 15 kilometers from their homes, therefore they had to go far. They advised locating a Stabilization Center (SC) in Nihla (Union Parishad) to cut traveling costs and make severe wasting treatment easier for the host community. Caregivers in both settings stressed the need for better access to key services for severely wasting children, in particular to reduce trip distances and increase caregiver convenience. | |
